# Two-Thumb or Two-Finger Technique in Infant Cardiopulmonary Resuscitation by a Single Rescuer? A Meta-Analysis with GOSH Analysis

**DOI:** 10.3390/ijerph17145214

**Published:** 2020-07-19

**Authors:** Chun-Yu Chang, Yueh-Tseng Hou, Yung-Jiun Chien, Yu-Long Chen, Po-Chen Lin, Chien-Sheng Chen, Meng-Yu Wu

**Affiliations:** 1 School of Medicine, Tzu Chi University, Hualien 970, Taiwan; paulchang1231@gmail.com; 2 Department of Emergency Medicine, Taipei Tzu Chi Hospital, Buddhist Tzu Chi Medical Foundation, New Taipei 231, Taiwan; 98311145@gms.tcu.edu (Y.-T.H.); yulong0129@gmail.com (Y.-L.C.); taipeitzuchier@gmail.com (P.-C.L.); holeyeye@yahoo.com.tw (C.-S.C.); 3 Department of Emergency Medicine, School of Medicine, Tzu Chi University, Hualien 970, Taiwan; 4 Department of Physical Medicine and Rehabilitation, Taipei Tzu Chi Hospital, Buddhist Tzu Chi Medical Foundation, New Taipei 231, Taiwan; jessica.kan.48@gmail.com

**Keywords:** cardiopulmonary resuscitation, two fingers, two thumbs, infant, chest compression

## Abstract

Out-of-hospital infant cardiopulmonary arrest is a fatal and uncommon event. High mortality rates and poor neurological outcomes may be improved by early cardiopulmonary resuscitation (CPR). The ongoing debate over two different infant CPR techniques, the two-thumb (TT) and the two-finger (TF) technique, has remained, especially in terms of the adequate compression depth, compression rate, and hands-off time. In this article, we searched three major databases, PubMed, EMBASE (Excerpta Medica database), and CENTRAL (Cochrane Central Register of Controlled Trials), for randomized control trials which compared the outcomes of interest between the TT and TF techniques in infant CPR. The results showed that the TT technique was associated with higher proportion of adequate compression depth (Mean difference (MD): 19.99%; 95%, Confidence interval (CI): 9.77 to 30.22; *p* < 0.01) than the TF technique. There was no significant difference in compression rate and hands-off time. In our conclusion, the TT technique is better in terms of adequate compression depth than the TF technique, without significant differences in compression rate and hands-off time.

## 1. Introduction

Out-of-hospital infant cardiopulmonary arrest is a fatal and uncommon event with a high mortality rate and poor neurological outcome [1,2,3]. The common etiology of cardiac arrest in infants is asphyxia. Early cardiopulmonary resuscitation with effective chest compressions and rescue ventilation may improve the clinical outcome. Current guidelines for infant cardiopulmonary resuscitation (CPR) recommend two chest compression techniques: the two-finger (TF) for a single rescuer and the two-thumb encircling (TT) chest compression for two or more rescuers [4,5]. In previous literature, the TT technique provided better chest compression depth than the TF in animals and manikin models [6,7,8,9]. However, there is a concern that the TT technique may elicit this advantage at the cost of longer time in switching from compression to ventilation during CPR, especially in a single rescuer. Although there is a lack of strong evidence to confirm this concern, the TT technique is currently not recommended for a lone rescuer. In our meta-analysis, we try to confirm this hypothesis by analyzing three major factors, namely chest compression rate, proportion of adequate compression depth, and hands-off time, to provide strong evidence for a difference between the two chest compression techniques in infant CPR performed by a single rescuer.

## 2. Method

### 2.1. Study Design

This systematic review and meta-analysis aimed to evaluate the effects of two different CPR techniques, the two-thumb technique and the two-finger technique, on infant manikin models. This study complies with the recommendations made by the Preferred Reporting Items for Systematic Review and Meta-analysis (PRISMA) statement [10].

### 2.2. Search Strategy

Two authors (Yung-Jiun Chien and Chun-Yu Chang) searched PubMed, EMBASE, and CENTRAL. Subject headings from PubMed, CENTRAL, and EMBASE (Mesh terms and Emtree terms) were used in combination with the title and abstract field tag or free-text words to facilitate searching. The following terms were used for searching: “cardiopulmonary resuscitation”, “heart arrest”, “heart massage”, “chest compression”, “infant”, “newborn”, “neonate”, “two-thumb”, “two-finger”, “two-thumb chest compression”, “two-finger chest compression”, “infant chest compression”, “newborn chest compression”, “infant cardiopulmonary resuscitation”, “newborn cardiopulmonary resuscitation”, “manikin”, and “mannequin”. We did not exclude studies by languages or geographical regions. Identified records were screened by titles, abstracts, and keywords. The reference lists of the identified records were used to manually search for relevant studies.

### 2.3. Eligibility Criteria and Risk of Bias in Individual Studies

All studies identified from electronic databases were screened and selected by two authors (Yung-Jiun Chien and Chun-Yu Chang) independently according to the inclusion criteria, with all of the following being met: (a) Randomized controlled trial (RCTs), either of parallel or crossover design; (b) comparison of conventional TT (with hands encircling the thorax) with TF; (c) studies reporting the outcomes of chest compression rate, proportion of adequate compression depth, and hands-off time; and (d) outcomes with sufficient information for meta-analysis. Two authors (Yung-Jiun Chien and Chun-Yu Chang) evaluated the methodological quality of all included studies by using the Risk of Bias 2 tool [11] for both the individually randomized, parallel-group trials and individually randomized, crossover trials. The third author (MYW) provided the consensus or discussion in the case of disagreements.

### 2.4. Data Extraction and Statistical Analysis

The relevant information was extracted by two authors (Yung-Jiun Chien and Chun-Yu Chang), including authors’ names, publication year, country, study design, number of participants, the expertise of participants, CPR duration, manikin model, manikin placement, and effect estimates. The effect estimates in each included study were calculated as mean difference (MD) and standard error (SE). The summary measurement (either MD or Hedges’ g, where suitable) with the 95% CI was then derived from pooling the effect of each included study using the inverse variance method with a random-effects model (DerSimonian–Laird estimator [12]). Heterogeneity (GOSH) was assessed by the Cochran Q statistic and quantified with the I^2^ statistic. Subgroup analysis was performed to evaluate whether the prespecified factors could account for the heterogeneity (i.e., locale, ventilation protocol, manikin model, expertise of the participants, and the placement of the manikin). Sensitivity analysis was used to test the robustness of the results. First, a leave-one-out analysis was performed by omitting one study at a time and reperforming meta-analysis to evaluate if the leave-one-out pooled summary measurement falls outside of the 95% CI of the overall summary measurement. Second, in the outcomes containing studies where the data input involved the assumption of correlation, we replaced the originally assumed correlation (i.e., the lowest observed) with the highest observed one among the other studies and zero and then reperformed meta-analysis. Third, a graphical display of study heterogeneity plot was generated [13], and three unsupervised learning algorithms, i.e., k-means clustering [14], density-based spatial clustering of applications with noise (DBSCAN) [15] and Gaussian mixture models [16], were used to identify the potential outliers. Alternatively, a Baujat plot was plotted to assist in identifying potential outlier(s) by visualizing the studies located at the right side of the plot that contribute considerably to the heterogeneity and/or summary measurement [17]. Meta-analysis was reperformed after excluding the potential outliers.

## 3. Results

### 3.1. Study Identification and Selection

**Figure 1:**
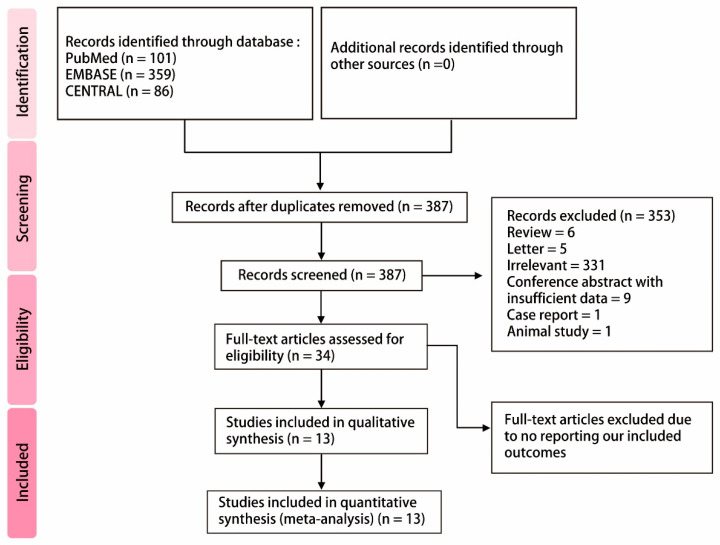
PRISMA flow diagram.

After searching three databases, including PubMed (*n* = 101), EMBASE (*n* = 359), and CENTRAL (*n* = 86), 546 articles were identified. A total of 159 articles were duplicates. The remaining studies were screened for eligibility; then, 353 articles were excluded due to not matching inclusion criteria. A total of 34 studies were assessed with full-text review; then, 21 studies were excluded due to not reporting outcomes of interest. Finally, 13 studies were included for meta-analysis. The detailed PRISMA flow diagram is shown in Figure 1.

### 3.2. Study Characteristics and Quality and Risk of Bias Assessment

**Figure 2:**
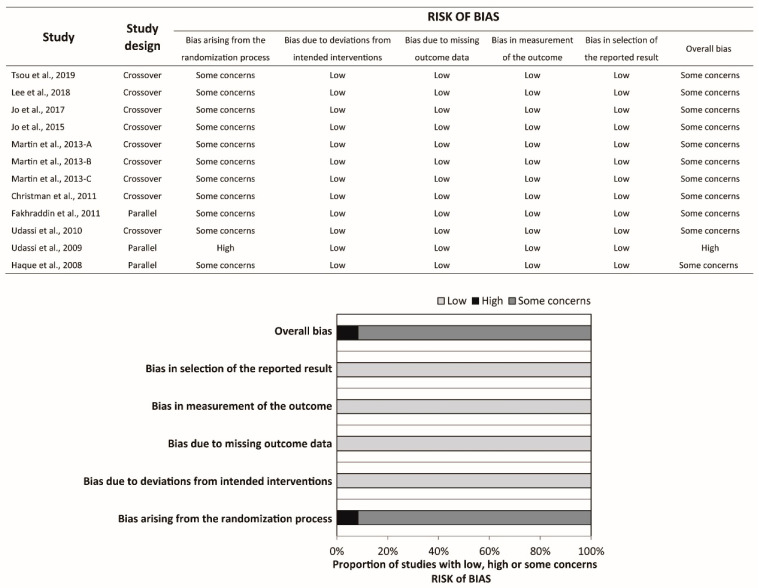
Risk of bias summary and graph of chest compression rate.

**Figure 3:**
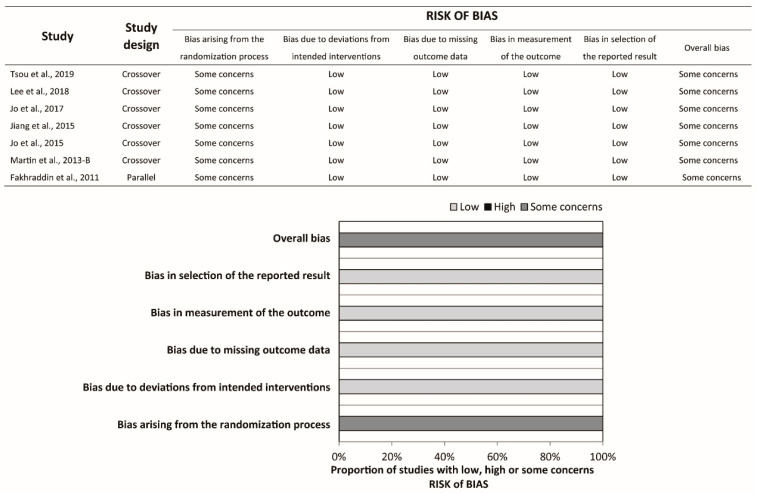
Risk of bias summary and graph of proportion of adequate compression depth.

**Figure 4:**
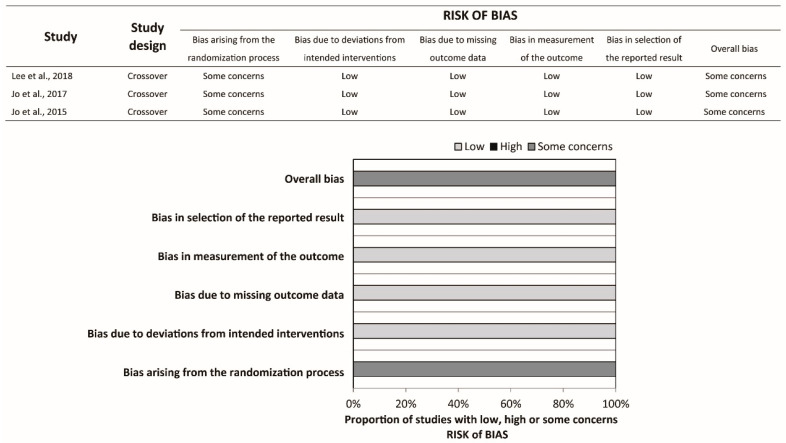
Risk of bias summary and graph of hands-off time.

Ten studies were crossover RCTs [18,19,20,21,22,23,24,25,26,27], whereas three were parallel RCTs [28,29,30]. In Haque et al.’s study [30], a total of 80 participants were randomly allocated to five groups, namely the infant TF, infant TT, child one-hand, child two-hand, and adolescent two-hand groups, with 16 participants in each group. Participants in each group were further randomized into two sequences, each starting with the compression-to-ventilation (C:V) ratio of 30:2 or 15:2. We extracted the relevant data in the infant TF and infant TT groups with C:V ratio 15:2 only. Hence, Haque et al.’s study should be regarded as parallel RCT in the present study. All studies compared the conventional TT technique to the TF technique. Participants were asked to stand at the head position while performing the TT technique (over-the-head TT) in two studies [20,21]. The CPR duration ranged from 1 to 5 min. Participants were asked to perform ventilation with the C:V ratio 30:2 in six studies and 15:2 in two studies, whereas the rest of the studies did not require the participants to perform ventilation. Participants with multiple areas of expertise were recruited in most of the studies, except for four studies where participants with a single area of expertise were recruited [19,20,21,22]. The risk of bias was assessed for each outcome, and the summary is available in Figure 2, Figure 3 and Figure 4 and Table 1.

### 3.3. Overall Summary Measurement

**Figure 5:**
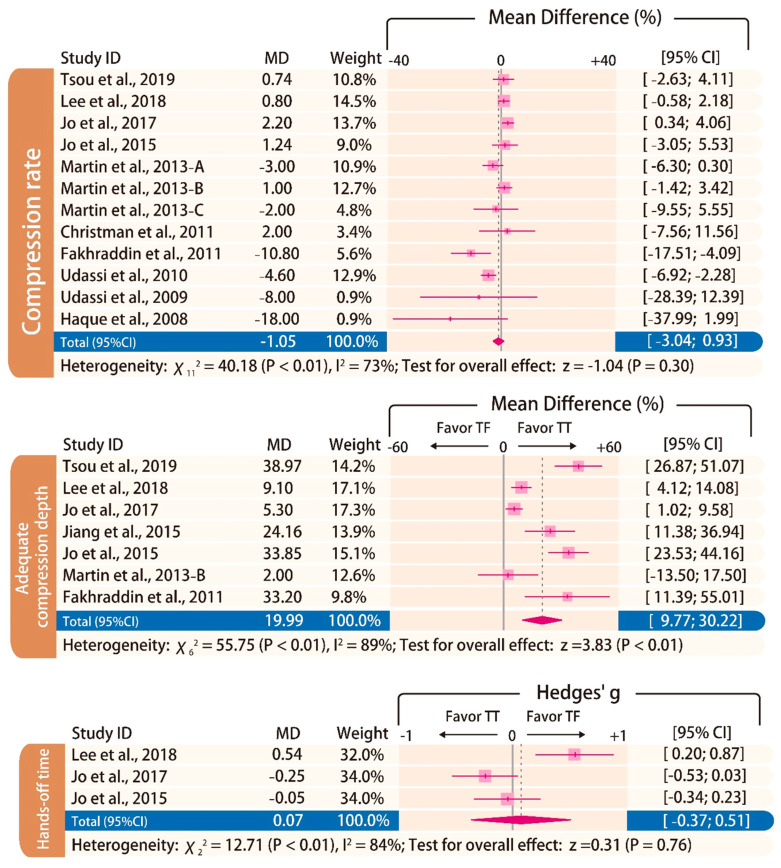
Forest plot of chest compression rate, proportion of adequate compression depth, and hand-off time.

There was no statistically significant difference in terms of chest compression rate (MD: −1.05/min; 95% CI: −3.04 to 0.93; *p* = 0.30). The proportion of adequate compression depth is higher using the TT technique than using the TF technique (MD: 19.99%; 95% CI: 9.77 to 30.22; *p* < 0.01). In addition, there was no statistically significant difference in terms of the hands-off time (Hedges’ g: 0.07; 95% CI: −0.37 to 0.51; *p* = 0.76; Figure 5). 

### 3.4. Subgroup Analysis of Chest Compression Rate

**Figure 6:**
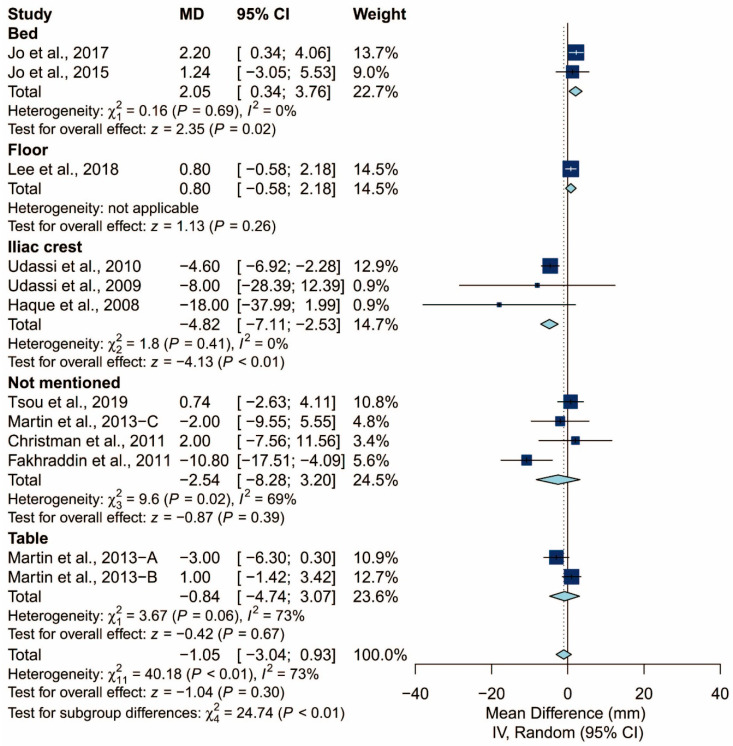
Subgroup analysis of chest compression rate grouped by locale.

**Figure 7:**
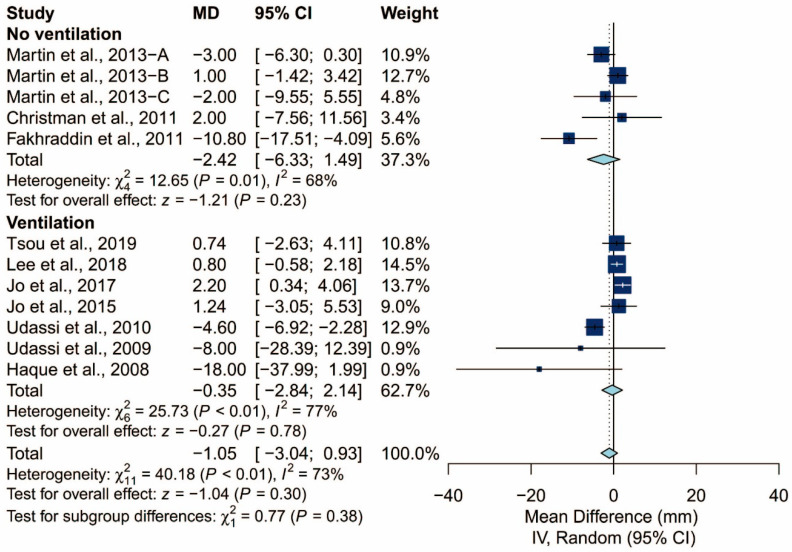
Subgroup analysis of chest compression rate grouped by ventilation protocol (yes/no).

**Figure 8:**
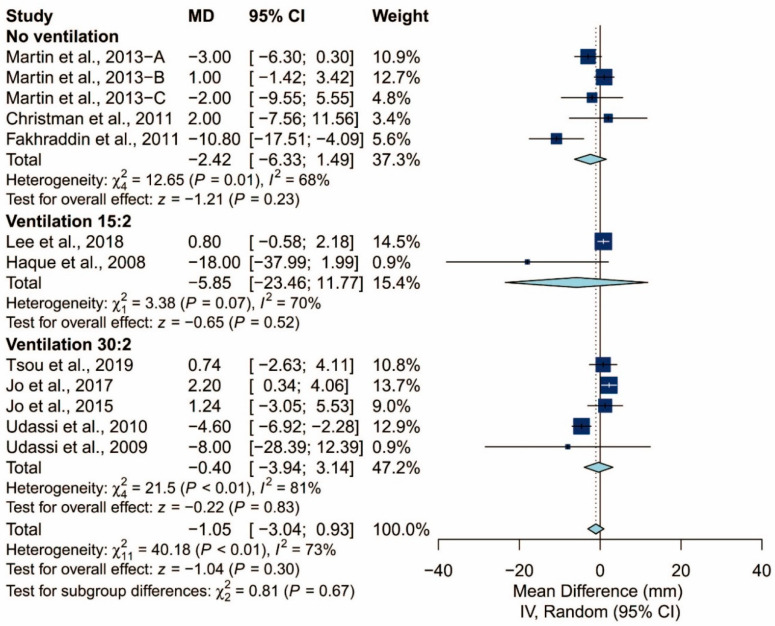
Subgroup analysis of chest compression rate grouped by ventilation protocol (15:2/30:2/no).

**Figure 9:**
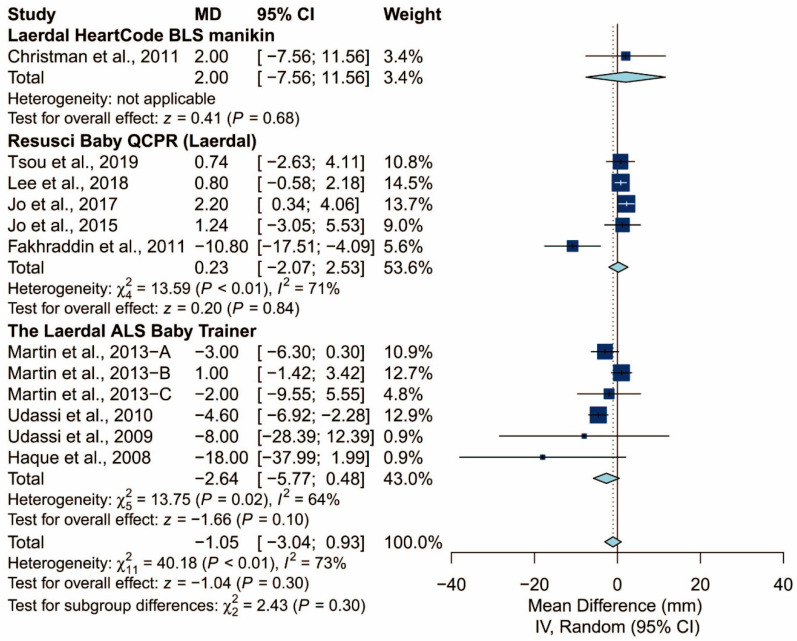
Subgroup analysis of chest compression rate grouped by manikin model.

**Figure 10:**
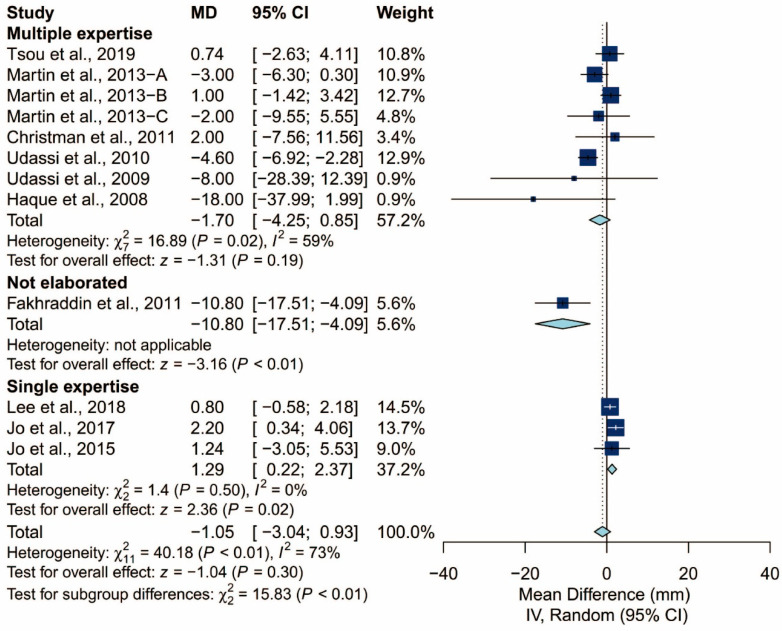
Subgroup analysis of chest compression rate grouped by expertise of the participants.

**Figure 11:**
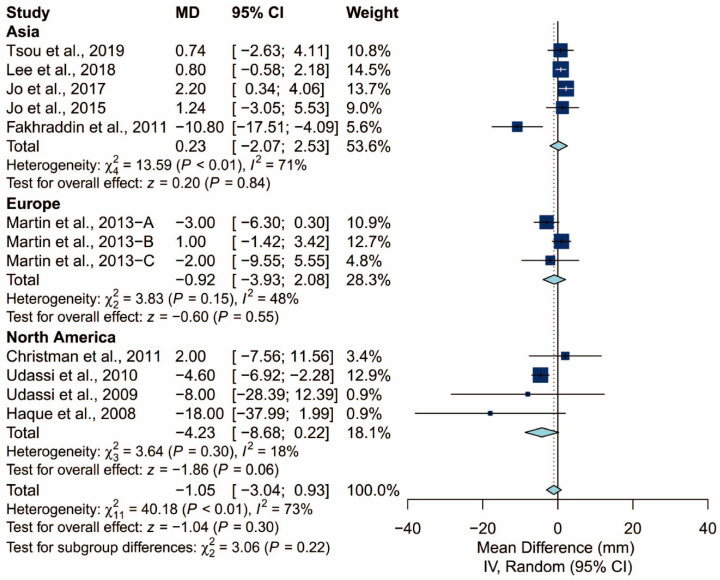
Subgroup analysis of chest compression rate grouped by manikin placement.

We found that the prespecified factors could not explain the heterogeneity observed in chest compression rate. First, I^2^ was 71% for studies conducted in Asia, 48% for those conducted in Europe, and 18% for those conducted in North America. Second, I^2^ was 68% for studies that did not require the participants to perform ventilation and 77% for those that did. When the latter was further grouped by the C:V ratio, heterogeneity remained high in both the 15:2 group (I^2^ = 70%) and the 30:2 group (I^2^ = 81%). Third, I^2^ was 71% and 64% for studies using Laerdal Resusci Baby QCPR and The Laerdal ALS Baby Trainer, respectively. Fourth, studies enrolling participants from single expertise showed low heterogeneity (I^2^ = 0%), but not those with multiple areas of expertise (I^2^ = 59%). Finally, studies where the manikin was placed on the bed (I^2^ = 0%) and height adjusted to the iliac crest (I^2^ = 0%) showed low heterogeneity but studies with the manikin on the table (I^2^ = 73%) did not. The detailed results can be seen in Figure 6, Figure 7, Figure 8, Figure 9, Figure 10 and Figure 11.

### 3.5. Subgroup Analysis of Proportion of Adequate Compression Depth

**Figure 12:**
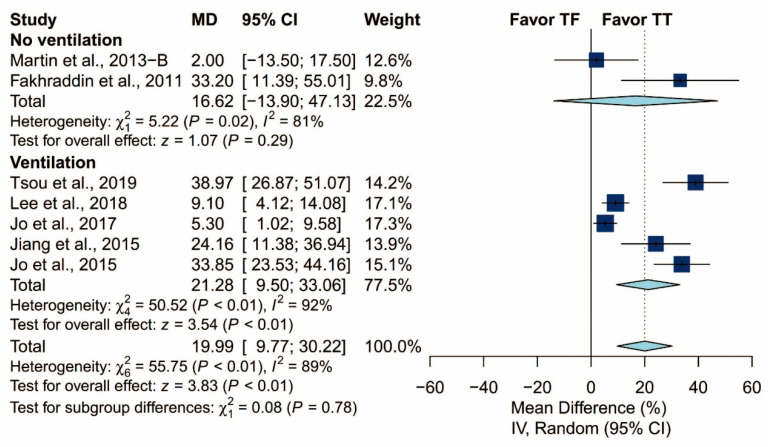
Subgroup analysis of the proportion of adequate compression depth grouped by ventilation protocol (yes/no).

**Figure 13:**
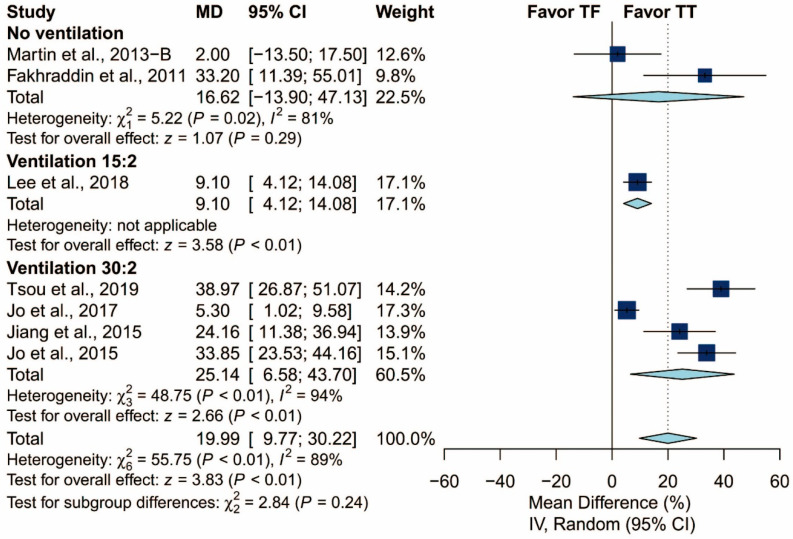
Subgroup analysis of the proportion of adequate compression depth grouped by ventilation protocol (15:2/30:2/no).

In subgroup analysis, only ventilation protocol was evaluated due to the relatively low number of included studies (Figure 12 and Figure 13). As in the previous results, the ventilation protocol did not explain the heterogeneity observed in the proportion of adequate compression depth. Heterogeneity was high in both groups of studies: those that did not require the participants to perform ventilation and those that did (I^2^ = 81% and I^2^ = 92%, respectively). 

### 3.6. Sensitivity Analysis of Chest Compression Rate

**Figure 14:**
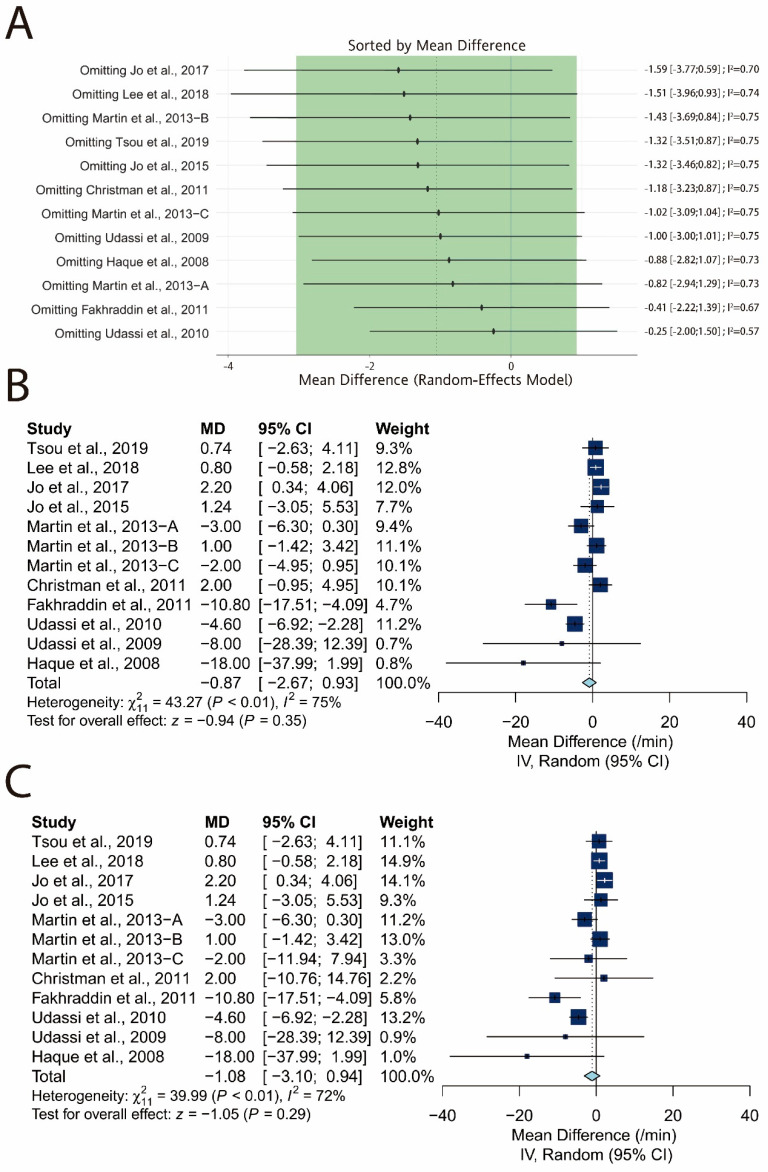
Sensitivity analysis of chest compression rate. (**A**) Leave-one-out analysis; (**B**) forest plot with the correlation set as the highest observed (0.95); (**C**) forest plot with the correlation set as 0.

**Figure 15:**
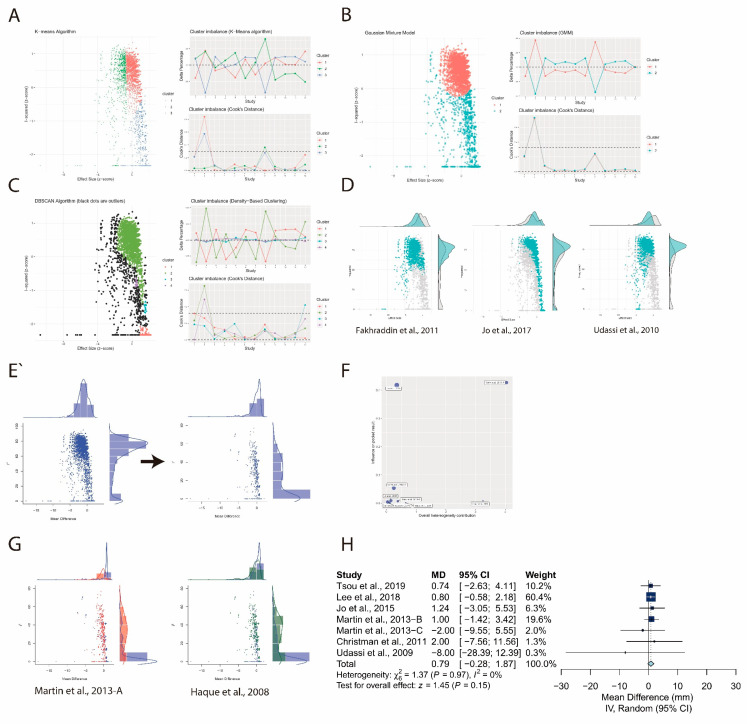
(**A**,**B**,**C**) Potential outliers identified by three unsupervised learning algorithms. (**D**) GOSH plots with the corresponding subsets including the potential outliers colored in green. (**E**) Left plot: the original GOSH plot; right plot: the GOSH plot after excluding the potential outliers in the first round. (**F**) Baujat plot containing the remaining studies after excluding the potential outliers in the first round. (**G**) GOSH plots with the corresponding subsets including the potential outliers identified from the Baujat plot colored in red and dark green. (**H**) Forest plot after excluding all the potential outliers.

First, leave-one-out analysis revealed that all the pooled estimates after omitting one study at a time still lie within the 95% confidence interval of the overall estimate (Figure 14A). Second, the lowest observed correlation of the TT and TF techniques among the other studies, which is 0.44, was assumed for two studies [25,26]. Similarly, we replaced the original correlation with the highest observed, which is 0.95, and zero and reperformed meta-analysis. The overall estimate remained nonsignificant after the correlation was replaced with the highest observed one (MD: −0.87/min; 95% CI: −2.67 to 0.93; *p* = 0.35; Figure 14B) and zero (MD: −1.06/min; 95% CI: −3.10 to 0.94; *p* = 0.29; Figure 14C). Third, three potential outliers [20,27,28] were identified in a similar fashion by three unsupervised learning algorithms (Figure 15A–C). The corresponding subsets including these potential outliers are shown in Figure 15D. However, because the GOSH plot remained heterogeneous (Figure 15E), we further explored the influence of each study by plotting the Baujat plot (Figure 15F). Two studies lay at the right side of the plot [24,30], and the corresponding subsets including these studies are shown in Figure 15G. We reperformed the meta-analysis after excluding the potential outliers, and the pooled estimate remained nonsignificant (MD: 0.79/min; 95% CI: −0.28 to 1.87; *p* = 0.15; Figure 15H) with low heterogeneity (I^2^ = 0%).

### 3.7. Leave-one-out Analysis of Proportion of Adequate Compression Depth and Hands-Off Time

**Figure 16:**
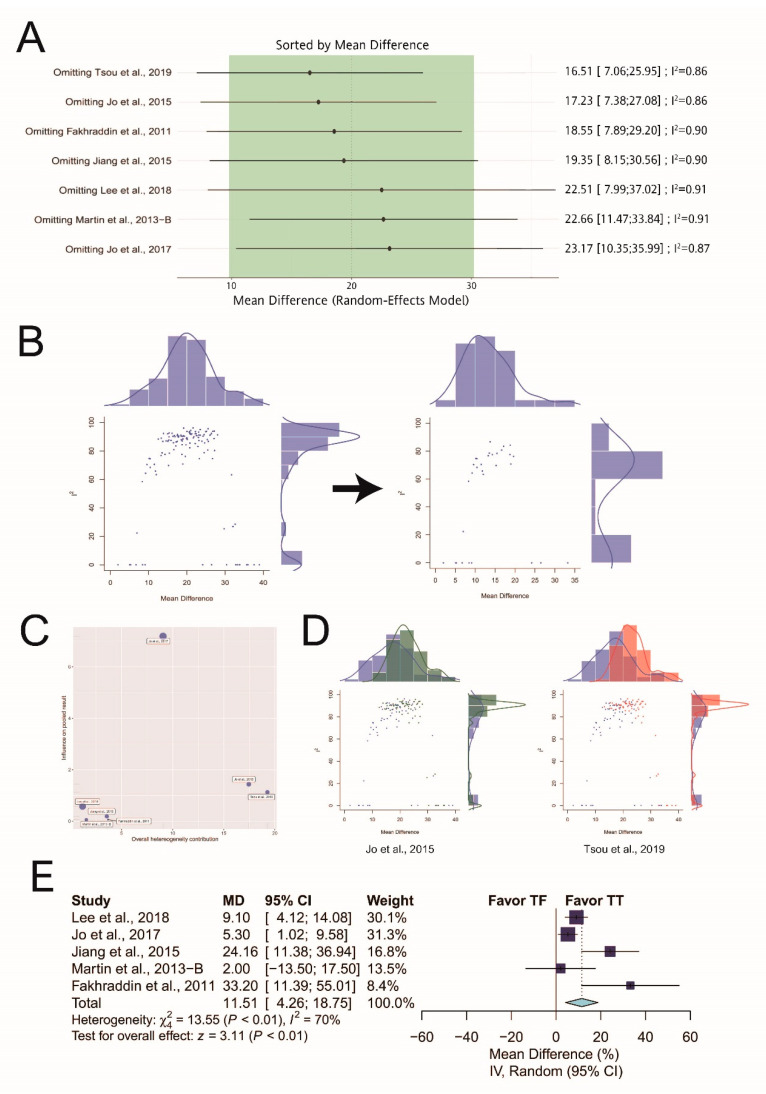
Sensitivity analysis of proportion of adequate compression depth. (**A**) Leave-one-out analysis. (**B**) The Baujat plot. (**C**) Left plot: the original GOSH plot; right plot: the GOSH plot after excluding the potential outliers identified from the Baujat plot. (**D**) GOSH plots with the corresponding subsets including the potential outliers identified from the Baujat plot colored in red and dark green. (**E**) Forest plot after excluding all the potential outliers.

First, leave-one-out analysis revealed that all the pooled estimates after omitting one study at a time still lie within the 95% confidence interval of the overall estimate (Figure 16A). Second, the Baujat plot showed two studies located at the right side of the plot [18,21] (Figure 16B). The corresponding subsets including these potential outliers were shown in Figure 16D. However, the GOSH plot remained heterogeneous after excluding the potential outliers (Figure 16C). We reperformed the meta-analysis after excluding the potential outliers, and the pooled estimate remained significant (MD: 11.51%; 95% CI: 4.26 to 18.75; *p* < 0.01; Figure 16E). In hands-off time, leave-one-out analysis was also performed for these outcomes. The results revealed that all the pooled estimates after omitting one study at a time still lie within the 95% confidence interval of the overall estimate in hands-off time.

## 4. Discussion

In our meta-analysis, the results showed that the TT technique generates significantly higher proportions of adequate compression depth than the TF technique. Our data were similar to those found in the previous studies. In Michael G. Millin et al.’s study [31], the TT technique showed greater compression depth and 36.91% more adequate compression depth than the TF technique. The compression depth is not only greater but also more consistent with the TT techniques. In addition, the subgroup analysis showed that the portion of adequate compression depth is better in TT than TF, regardless of the ventilation protocol (15:2 or 30:2). These results may be explained by the fact that the TF technique was relatively unsteady and more easily caused fatigue during CPR, especially in shifting between ventilation and chest compression. 

Three major challenges have been emphasized in the current guidelines for HP-CPR: shallow chest compressions, excessive compression rates, and prolonged duty cycles. In Haque et al.’s study [30], the authors revealed a trend of higher compression rate in the infant-sized manikin as compared with the adult manikins due to smaller compression displacement required. Excessive compression rates prohibited the chest wall from complete recoil, leading to decreased venous return and cardiac output. In our results, there was no significant difference in compression rates between the TT and TF techniques. This result remained the same in different manikin models and under different ventilation protocols (i.e., a C:V ratio of 15:2, a C:V ratio of 30:2, or no ventilation at all) in our subgroup analysis. 

Hands-off time is another concern when performing the TT technique during infant CPR by a single rescuer. Although the present study suggested that there was no significant difference in hands-off time between the two techniques, the relatively low number of studies included in this outcome decreased our confidence to make such a conclusion. While most of the studies reported longer hands-off time in the TT technique [19,32,33,34], several modified techniques for infant CPR have been proposed to improve hands-off time and maintain adequate compression depth. In Jo et al.’s studies [20,21], they proposed that the rescuers performed the TT technique at the head of the manikin (and hence the name over-the-head two-thumb encircling technique (OTTT)). Moving the rescuer from the side to the head position of the manikin can shorten the time between chest compression and ventilation. The authors revealed that the mean hands-off time of the OTTT technique was similar to that of the TF technique (7.6 ± 1.1 vs. 7.9 ± 1.3 s, *p* = 0.885) [20]. In Jacek Smereka et al.’s studies [34,35,36,37,38,39], a new two-thumb chest compression technique (nTTT) was promoted, which consisted of the two thumbs directed at the angle of 90° to the chest. This method may provide the same chest compression force as TT and get the same full recoil and hand-off time as TF. In these studies, the performance of nTTT is comparable to the recommendations laid out in the current guidelines in terms of compression depth, hands-off time, and ventilation quality. On the other hand, the “knocking-fingers” chest compression technique (KF) proposed by Jung et al. [33] is a novel chest compression technique that uses the tip of the thumb against the palmar side of the index finger with flexion of the proximal interphalangeal joint and the distal interphalangeal joint. The KF technique shortened the total hands-off time (median: 70 vs. 72 s) while maintaining the proportion of adequate compression depth as compared with the TF technique. Further investigation is required to confirm the effects of the novel chest compression techniques.

Several limitations were noted in this study. First, although the proportion of adequate compression depth is better in TT than TF, the results may not effectively reflect the coronary perfusion pressure, which is the most effective parameter for chest compression quality. Second, although there was no significant difference in compression rates between the TT and TF techniques, it may be explained that the CPR duration was shorter than in real infant cardiac arrest. The fatigue in the CPR rescuer would be more significantly detected in the TT and TF techniques during a prolonged CPR course. However, the longest CPR duration is only five minutes in our included articles. Third, all the included studies were tested on a manikin model. Finally, there are few studies focused on our outcomes of interest, and there are especially few studies focused on hands-off time. In the future, large randomized clinical trials are necessary to confirm our results.

## 5. Conclusions

In conclusion, our results indicate that the TT technique is superior to the TF technique in terms of adequate compression depth, without significant difference in compression rate and hands-off time. 

## Figures and Tables

**Table 1:** The detailed characteristics of the included studies.

Study	RCT Design	Patient Number	Intervention	Comparison	CPR Time (min)	Ventilation	Manikin	Manikin Placement	Participants
Tsou et al., 2019 [18]	crossover	42	TT	TF	2	30:2	Resusci Baby QCPR (Laerdal)	Not mentioned	EMTs, RNs
Lee et al., 2018 [19]	crossover	37	TT	TF	2	15:2	Resusci Baby QCPR (Laerdal)	Floor	Physicians
Jo et al., 2017 [20]	crossover	48	OTTT	TF	2	30:2	Resusci Baby QCPR (Laerdal)	Bed	Medical students
Jiang et al., 2015 [22]	crossover	27	TT	TF	5	30:2	Resusci Baby QCPR (Laerdal)	Iliac crest	Physicians
Jo et al., 2015 [21]	crossover	46	OTTT	TF	2	30:2	Resusci Baby QCPR (Laerdal)	Bed	RNs
Martin et al., 2013-A [24]	crossover	22	TT	TF	2	No	The Laerdal ALS Baby Trainer	Table	Physicians, RNs, resuscitation officers
Martin et al., 2013-B [23]	crossover	40	TT	TF	1.5	No	The Laerdal ALS Baby Trainer	Table	Resuscitation officer, physicians, RNs, operating room practitioner, paramedics
Martin et al., 2013-C [25]	crossover	35	TT	TF	1	No	The Laerdal ALS Baby Trainer	Not mentioned	Resuscitation officers, physicians, RNs
Christman et al., 2011 [26]	crossover	25	TT	TF	1	No	Laerdal HeartCode BLS manikin	Not mentioned	Physicians, RNs
Fakhraddin et al., 2011 [28]	parallel	40	TT	TF	5	No	Resusci Baby QCPR (Laerdal)	Not mentioned	PALS providers
Udassi et al., 2010 [27]	crossover	34	TT	TF	2	30:2	The Laerdal ALS Baby Trainer	Iliac crest	Faculty, physicians, RNs, medical/nursing students, pharmacists, RTs, NPs
Udassi et al., 2009 [29]	parallel	32	TT	TF	5	30:2	Resusci Baby QCPR (Laerdal)	Iliac crest	RNs, medical students, physicians, faculty, others
Haque et al., 2008 [30]	parallel	32	TT	TF	5	15:2	The Laerdal ALS Baby Trainer	Iliac crest	Faculty, physicians, RNs, medical/nursing students, RTs, OTs

TT: two-thumb technique; OTTT: over-the-head two-thumb technique; TF: two-finger technique; CPR: cardiopulmonary resuscitation; EMT: emergency medical technician, RN: registered nurse; NP: nurse practitioner; PALS: pediatric advanced life support; RT: respiratory therapist; OT: occupational therapist.
